# Protein cargo in extracellular vesicles as the key mediator in the progression of cancer

**DOI:** 10.1186/s12964-023-01408-6

**Published:** 2024-01-10

**Authors:** Klára Hánělová, Martina Raudenská, Michal Masařík, Jan Balvan

**Affiliations:** 1 Department of Pathological Physiology, Faculty of Medicine, Masaryk University, Kamenice 5, Brno, CZ-625 00 Czech Republic; 2 Department of Physiology, Faculty of Medicine, Masaryk University, Kamenice 5, Brno, CZ-625 00 Czech Republic; 3 BIOCEV, First Faculty of Medicine, Charles University, Prumyslova 595, Vestec, CZ-252 50 Czech Republic

**Keywords:** Extracellular vesicles, Exosomes, Exosomal proteins, Cancer, Tumour microenvironment, Cancer-associated fibroblasts, Angiogenesis, Cell death, Immune evasion, Metastasis, Therapy resistance, Biomarkers

## Abstract

Exosomes are small vesicles of endosomal origin that are released by almost all cell types, even those that are pathologically altered. Exosomes widely participate in cell-to-cell communication via transferring cargo, including nucleic acids, proteins, and other metabolites, into recipient cells. Tumour-derived exosomes (TDEs) participate in many important molecular pathways and affect various hallmarks of cancer, including fibroblasts activation, modification of the tumour microenvironment (TME), modulation of immune responses, angiogenesis promotion, setting the pre-metastatic niche, enhancing metastatic potential, and affecting therapy sensitivity and resistance. The unique exosome biogenesis, composition, nontoxicity, and ability to target specific tumour cells bring up their use as promising drug carriers and cancer biomarkers. In this review, we focus on the role of exosomes, with an emphasis on their protein cargo, in the key mechanisms promoting cancer progression. We also briefly summarise the mechanism of exosome biogenesis, its structure, protein composition, and potential as a signalling hub in both normal and pathological conditions.

Video Abstract

**Supplementary Information:**

The online version contains supplementary material available at 10.1186/s12964-023-01408-6.

## Introduction

Extracellular vesicles (EVs) belong to the group of heterogeneous membranous structures derived from all cell types, even those pathologically altered [1]. Depending on the cell type they originated from, EVs can contain various sets of specific proteins. Based on their size and biogenesis, EVs can be divided into three main groups: apoptotic bodies, ectosomes, and exosomes. Apoptotic bodies are released by cells that have undergone apoptosis and are 1,000–5,000 nm in diameter. Ectosomes, formed from plasma membrane outward budding (ectocytosis), are 150–1,000 nm in diameter and include vesicles such as oncosomes and microvesicles [2, 3]. Exosomes are generated by the endolysosomal system by exocytosis of intraluminal vesicles (ILVs) formed within the multivesicular bodies (MVBs) and are, typically, 30–150 nm in diameter [4].

EVs are nanosized particles formed by phospholipid membrane, that carry various sets of proteins, lipids, nucleic acids, glycans, and others that reflect the content of their cell of origin [5]. EVs are essential mediators of intercellular communication and delivery vehicles of molecular signals through the extracellular space. They play crucial roles in the homeostasis of healthy tissues and the progression of pathological states, including cancer, by stimulating cell proliferation, angiogenesis, metastasis, and other tumour-promoting processes. Via their content, EVs can regulate various signalling pathways [6, 7]. Cells in pathological conditions secrete large quantities of various EVs into body fluids, reflecting the organism's disease state. Therefore, these disease-specific EV surfaces and contents could be used as sensitive biomarkers and have great potential as liquid biopsy agents for various diseases [8–10].

To understand how different types of EVs affect cancer development, accurate nomenclature is essential. The International Society for Extracellular Vesicles (ISEV) proposed the Minimal Information for Studies of Extracellular Vesicles (“MISEV”) guidelines in 2014, which were updated four years later [11, 12]. The recommendation is to use the term “extracellular vesicle” as a “generic term for particles naturally released from the cell that are delimited by a lipid bilayer and cannot replicate” [11, 13]. The term exosome was initially used to refer to a membrane vesicle released by reticulocytes during their maturation [4, 14]; now it is used to describe MVB-origin EVs [13]. In this review, we are mainly focused on cancer-associated exosomes and their protein content, and discuss other EVs in comparison if relevant.

## The structure and content of exosomes

The secretion of exosomes was originally proposed as a mechanism that serves to eliminate unnecessary proteins from the cell [15]. However, in the 1990s, it was suggested that exosomes could play a role in intracellular communication, especially if connected to immune responses and cancer [16, 17]. This concept was supported later in 2007, when mRNAs and miRNAs were shown to be present in exosomes in their functional form and thus able to alter cell behaviour. This RNA was called “exosomal shuttle RNA” (esRNA) [18]. Besides esRNAs, exosome content includes several molecules, such as proteins, lipids, other nucleic acids, and metabolites, highly reflecting the identity and molecular state of their cell of origin [19]. Approximately 4400 proteins, 194 lipids, 1639 mRNAs and 764 miRNAs were identified in exosomes from different cell types, which points to their potential functional diversity and complexity [20, 21].

Exosomal cargo is protected from enzymatic degradation as it is encapsulated within the lipid bilayer of exosomes. Exosomal proteins can maintain the native conformation and functionality (for example, exosomal phosphoproteins were stable over a storage period of 5 years [22]). This makes them useful for the transfer of intact and functional proteins between cells. The protein content of the exosomes can directly influence the behaviour of the targeted cells, their microenvironment, and cell-to-cell communication [23, 24] (mRNA must be translated to have some influence). Therefore, exosomal proteins can provide better interpretable and more accurate information about the nature of communication in the tumour microenvironment (TME), disease progression and the degree of TME transformation. The selection of exosome cargo is not a random process. It requires the involvement of complex sorting mechanisms [19]. The state of the cell that produced these EVs can influence the content and biogenesis of these vesicles by various molecular signals. For example, tumour cells in a state of hypoxia secrete EVs that help to enhance angiogenic and metastatic potential [25]. Exosomes are potentially highly attractive objects for proteomic research as they are highly enriched in membrane and other proteins, which are poorly represented in most purely proteomic studies for their low concentrations or biophysical properties in isolated samples. Additionally, the presence of a specific set of proteins enables the recognition of specific cell types in the investigated sample and even sheds light on changes in cellular behaviour [19].

As mentioned above, exosomes are membrane vesicles composed of a hydrophilic core surrounded by a lipid bilayer which express, on the surface, various ligands, receptors and other bioactive molecules derived from the source cells [26]. The key components of the lipid membrane are phosphatidylcholines (PC), phosphatidylethanolamines (PE), phosphatidylinositols (PS-PI), sphingomyelins (SM), gangliosides, ceramides, cholesterol, or diacylglycerols (DAG) [27–30]. The ratio of lipids in the exosomal membrane differs slightly from the composition of these lipids in the plasma membrane. In addition, exosome membrane “flip-flop” transitions are more common than in the plasma membrane due to the lack of flippases [31]. Consequently, some EVs, such as apoptotic exosome-like vesicles [32] but also exosomes secreted by cancer cells, can expose phosphatidylserine (PS) on the surface [24, 33, 34]. Besides the structural role of lipids in the exosomal membrane, they are essential for exosome formation and release [35]. The lipid content of the exosomal core is mainly represented by phospholipids, glycolipids and free fatty acids, in contrast to microvesicles, which are enriched in ceramides and sphingomyelins [36]. Exosomes also carry surface receptors, adhesion molecules (ICAMs), integrins, tetraspanins (CD9, CD63, CD81, CD82, CD53, and CD37) and other transmembrane or surface proteins. The protein content of exosomes is represented by the cytosolic and cytoskeletal proteins (β-actin), enzymes (GTPases, proteases), heat shock proteins (HSP60, HSP70, HSP90), cytokines, endosome-associated proteins (Alix, Tsg101, Rab proteins), or oncoproteins [37]. Some of these molecules, such as tetraspanins CD9, CD63, and CD81, or Tumour susceptibility gene 101 (Tsg101) and ALG-2-interacting protein X (Alix), are considered exosomal markers [38–40].

## Exosome biogenesis and trafficking

**Fig. 1:**
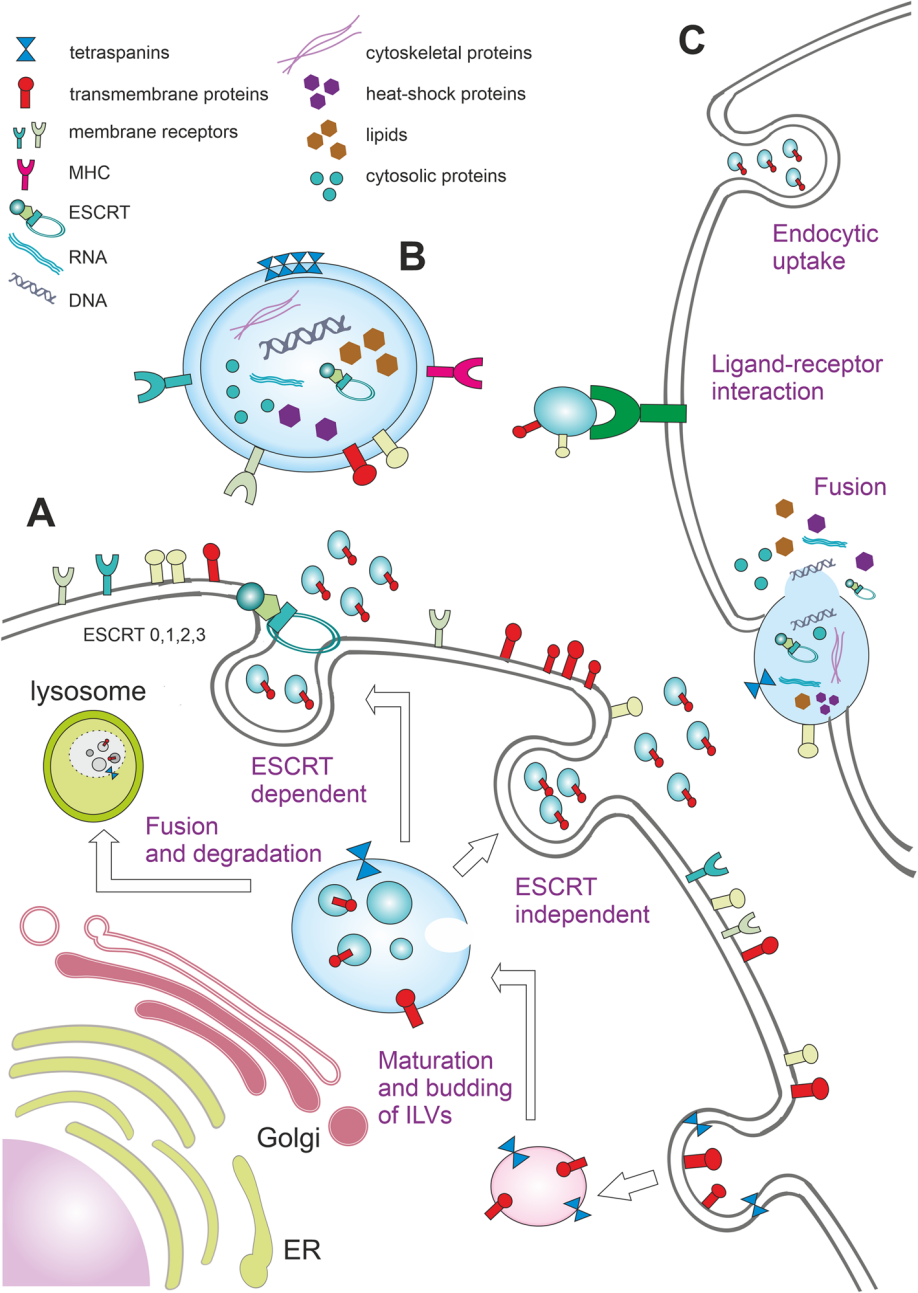
Exosome biogenesis, release, structure, content, and uptake by recipient cells. **A** Exosomes originate from the invagination of the plasma membrane, forming an early endosome. The inward budding of the endosome creates ILVs within the MVB (late endosome), which can be either degraded in lysosome, or secreted into the extracellular space as exosomes via ESCRT-dependent, or ESCRT-independent pathway, in which case lipid domains are involved. **B** Exosomes are formed by the lipid bilayer with integrated bioactive molecules at its surface, such as cell adhesion molecules, tetraspanins, cytokine or MHC receptors, and integrins. The exosome content comprises cytosolic and cytoskeletal proteins, enzymes, ESCRT components, various types of nucleic acids, and lipids. **C** The exosome uptake can be provided non-specifically by endocytosis and simple fusion with the plasma membrane or specifically by the ligand-receptor interaction

The biogenesis of exosomes consists of many complex steps and is regulated by various intra- and extracellular signals. Firstly, the biogenesis mechanism involves the formation of an early endosome, which is derived from the plasma membrane by endocytosis. Secondly, the inward budding of the early endosome creates small intraluminal vesicles (ILVs), giving rise to multivesicular endosomes (MVEs), also called multivesicular bodies (MVBs) or late endosomes [41]. Finally, these MVBs fuse with the plasma membrane and release ILVs into the extracellular space as exosomes [42] (see Fig. 1). This unique process differentiates exosomes from other EVs, such as apoptotic bodies, oncosomes, or necrotic blebs. Exosome biogenesis usually requires the endosomal sorting complex required for transport (ESCRT), although an ESCRT–independent pathway has also been identified [43]. Exosome biogenesis allows the regulation of protein quality, as it enables cells to retrieve proteins from the plasma membrane selectively. Released exosomes play a role in a wide range of processes, such as signal and molecular transmission to other cells, or extracellular matrix (ECM) remodelling [44].

The ESCRT machinery comprises around 30 proteins assembled into 5 functional subcomplexes. The ESCRT-0 sequesters ubiquitinated cargo, ESCRT-I/II/III are implicated in ILVs budding, and the VPS4 complex is responsible for membrane scission. The ESCRT machinery enables both the selection of endocytic cargo incorporated into ILVs and the formation of ILVs themselves by membrane remodelling and scission. The cargo selection is mediated by the accessory Alix/Syntenin/Syndecan complex, which also directs the biogenesis of ILVs [45, 46]. The formation of MVBs driven by ESCRT is critical not only for exosome biogenesis but also for lysosomal degradation via targeting ubiquitylated proteins [47].

On the other hand, the ESCRT–independent pathway is driven by lipids and associated proteins like tetraspanin CD63 in a lipid-mediated process dependent on the self-organising of lipid and cargo domains. The presence of ceramide, lysophospholipid and glycosphingolipid molecules on the limiting membrane induces spontaneous budding within the MVBs to produce ILVs and tetraspanin sorting into ILVs [48–50]. Rab GTPase Rab31 drives and controls exosome biogenesis independent of ESCRT differently than ceramides and tetraspanins. Rab31, which enables ILV formation, inactivates Rab7 and suppresses the fusion of MVBs with lysosomes, thereby promoting the secretion of exosomes. Therefore, Rab31 represents the key checkpoint for exosome biogenesis and determines its fate by balancing with Rab7 [51, 52].

### Exosome secretion, uptake and cargo delivery

The microtubule network is responsible for the transport of MVBs towards the plasma membrane. It requires altered actin polymerisation near the plasma membrane, followed by actomyosin cytoskeleton contraction [53]. The fusion of MVBs with the plasma membrane was found to be mediated by SNARE (SNAP receptor) complex formed by vesicular v-SNARE and target membrane t-SNARE [54]. After the contact of these two membranes, the SNARE complex overcomes the fusion energy barrier due to its association with the V-ATPase subunit V0. Exosomes are also rich in various Rab GTPases, which are believed to regulate membrane trafficking and secretion, Rab4, Rab5, Rab11 and Rab27 in particular [55].

The mechanism of exosome release is classified as constitutive or inducible, depending on the cell of origin. The constitutive secretion pathway is provided by Rab GTPases, heterotrimeric G-protein, and protein kinase D [56]. The inducible secretion is mediated by various types of physical, biological, and chemical stimuli, such as low pH, DNA damage, change in extracellular ATP levels, hypoxia, and increased intracellular Ca^2+^. For example, under the influence of hypoxia, cardiomyocytes release an increased number of exosomes [57]. Exosome secretion also depends on lipid mediators, like diacylglycerol, and is regulated by p53 via TSAP6 (Tumour suppressor activated pathway-6) [47, 58]. However, fusion with the plasma membrane is not the only fate MVBs can undergo. MVBs can also be directed to lysosomes, where their content is degraded and not secreted from the cell. By fusion with autophagosomes, MVBs can give rise to amphisomes, which may fuse with the plasma membrane and release their content extracellularly or can be degraded in lysosomes [45, 59].

The specific targeting towards recipient cells depends on the composition of the exosome surface. For instance, complex lipids influence exosome targeting in cancer cells. Sphingomyelin-enriched melanoma-derived exosomes exhibit enhancement in targeting within the TME [28]. Similarly, exosomes derived from glioblastoma cells enriched with phosphatidylethanolamine mainly target glioblastoma cells [60]. Exosomes mediate cell-to-cell communication, both locally and systemically, and may pass multiple uptake and release cycles allowing them to access several layers of tissues of multiple organs, including the liver, kidney, lung, pancreas, spleen, colon, ovaries and last, but not least brain [61, 62]. In contrast, large EVs ( > 200µm) are predominantly accumulated in bones, liver and lymph nodes, which points to the fact that the transport of exosomes is also influenced by their size [63].

The internalisation of exosomes into the recipient cell can be provided through a non-specific process, such as endocytosis, including caveolae-dependent and clathrin-dependent endocytosis, pinocytosis and phagocytosis [64], or specifically by receptor-dependent pathway [53]. The specific targeting is proposed to be mediated by many proteins localised on the cell surface, including integrins, lectins, and T-cell immunoglobulin, or mucin domain-containing protein 4 (Tim4) [65, 66]. Tim4 is a transmembrane protein expressed on macrophages, which specifically binds the phosphatidylserine displayed on the EV surface [67]. Nevertheless, the uptake route may be more dependent on the recipient cell type than on the exosomes themselves [53]. However, exosomes can transmit information not only by integrating into the recipient cells but also by acting at the cell surface, for example, during an immune response. In this case, exosomes harbouring major histocompatibility complex (MHC) can activate related T-cell receptors on T-lymphocytes [68].

Interestingly, exosome release and uptake by cancer cells can be highly influenced by a low pH in the TME. On the metastatic melanoma cells was shown, that compared to buffered conditions, low pH conditions increased both, exosome release and uptake [69, 70]. The study by Parolini et al. also showed a change in exosome membrane rigidity in a low pH in association with the increased amount of N-acetylneuraminylgalactosylglucosylceramide (GM3) and sphingomyelin (SM), which are known to be parts of membrane microdomains, also known as lipid rafts. This elucidates the increased fusion capacity of exosomes in a low pH, as sphingomyelin modulates the efficiency of membrane fusion [69, 71]. In addition, the amount of EVs and their content can be influenced by the autophagy machinery [34, 72]. The secretion of pro-angiogenic EVs during hypoxia is dependent on the autophagy-related protein GABARAPL1 [73]. The starvation of cancer cells significantly altered the composition of the protein content of phosphatidylserine-positive EVs (PS-EVs) produced by these cells. Starvation increased the exosomal abundance of matrix metalloproteinase 13 (MMP13), which can promote angiogenesis, and decreased the abundance of periostin and regucalcin (RGN) in PS-EVs [34]. Secreted POSTN can promote cancer stemness in head and neck cancer, and RGN promotes dormancy in cancer cells [74, 75].

## Exosomal proteins as signal molecules

Recently, a growing body of evidence has emerged to support the involvement of exosomes in the regulation of a variety of signalling pathways, including WNT and KRAS signalling [76] or PI3K/AKT or MAPK/ERK pathways [77]. These pathways can influence stem cell maintenance, cell differentiation, tissue repair and regeneration processes. Consequently, exosomes have important signalling roles in affecting their surroundings, the recipient cells or even distant environment [78]. Exosomal proteins can contain growth factors and cytokines that trigger signalling pathways promoting cell growth, proliferation, or angiogenesis. For example, exosomes containing EGFR influence the liver microenvironment, facilitating the metastasis of gastric cancer to the liver [79]. Proteins involved in drug efflux transport, like P-glycoprotein, can be packaged within exosomes, protecting cells from chemotherapy agents [80]. Some types of exosomes can carry hormonal signals, such as steroid hormones, which have been detected in urinary exosomes [81]. Exosomal proteins can also deliver activation signals to recipient cells. Immune cells can be stimulated by exosomes from antigen-presenting cells (APCs) like macrophages and dendritic cells (DCs). These exosomes carry MHC molecules with antigens on their surface. Their uptake by the T-cell receptor (TCR) of specific T-cells subsequently leads to T-cell activation [82]. Exosomes can contribute to inflammatory signalling by transporting pro-inflammatory or anti-inflammatory cytokines [83] and may also carry immunomodulatory proteins, such as programmed death-ligand 1 (PD-L1), which can suppress the activity of cytotoxic T-cells, leading to immune evasion by tumour cells [84].

Many proteins identified in exosomes including lactate dehydrogenase A (LDHA), annexin A1/2 (ANXA1/2), or HSP90, are known to be mutated in multiple cancer types [21]. Specifically, exosomal extracellular matrix protein 1 (ECM1) was found to promote progression and even metastatic invasion in most cancers. Thus, ECM1 is thought to be an indicator of increased metastatic potential of tumour cells and was linked to poor prognosis [85, 86]. Another protein identified in exosomes, alpha-2-HS-glycoprotein (AHSG), was found to promote breast cancer progression and was associated with the risk of colorectal carcinoma and non-small cell lung cancer (NSCLC) [87–89].

Some membrane proteins were found to possess therapeutic properties, but those are achievable only if these proteins remain in their native, or close to native conformation. Fortunately, exosomes can act as scaffolds for membrane proteins, thus they can be maintained in their native state [90]. For example, TNF-related apoptosis-inducing ligand (TRAIL) located on the exosomal surface could deliver apoptosis signals to tumour cells and promote apoptosis [91].

EVs are, among other processes, involved in unconventional protein secretion (UPS) [92]. This mechanism includes proteins lacking a signal sequence in their gene, which enables them to enter the ER—Golgi apparatus (GA) conventional pathway, such as interleukin-1β, fibroblast growth factor 2 (FGF-2), or bacterial enzymes [93, 94]. These proteins are essential molecules that function in cell signalling, immune modulation and many other extracellular pathways [95, 96]. The fact that EVs are present in several body fluids enables them to trigger biological responses in distant locations, such as metastatic sites, and supports their potential as biomarkers and therapeutic vehicles [25, 97, 98].

## Exosomal proteins in cancer progression

**Fig. 2:**
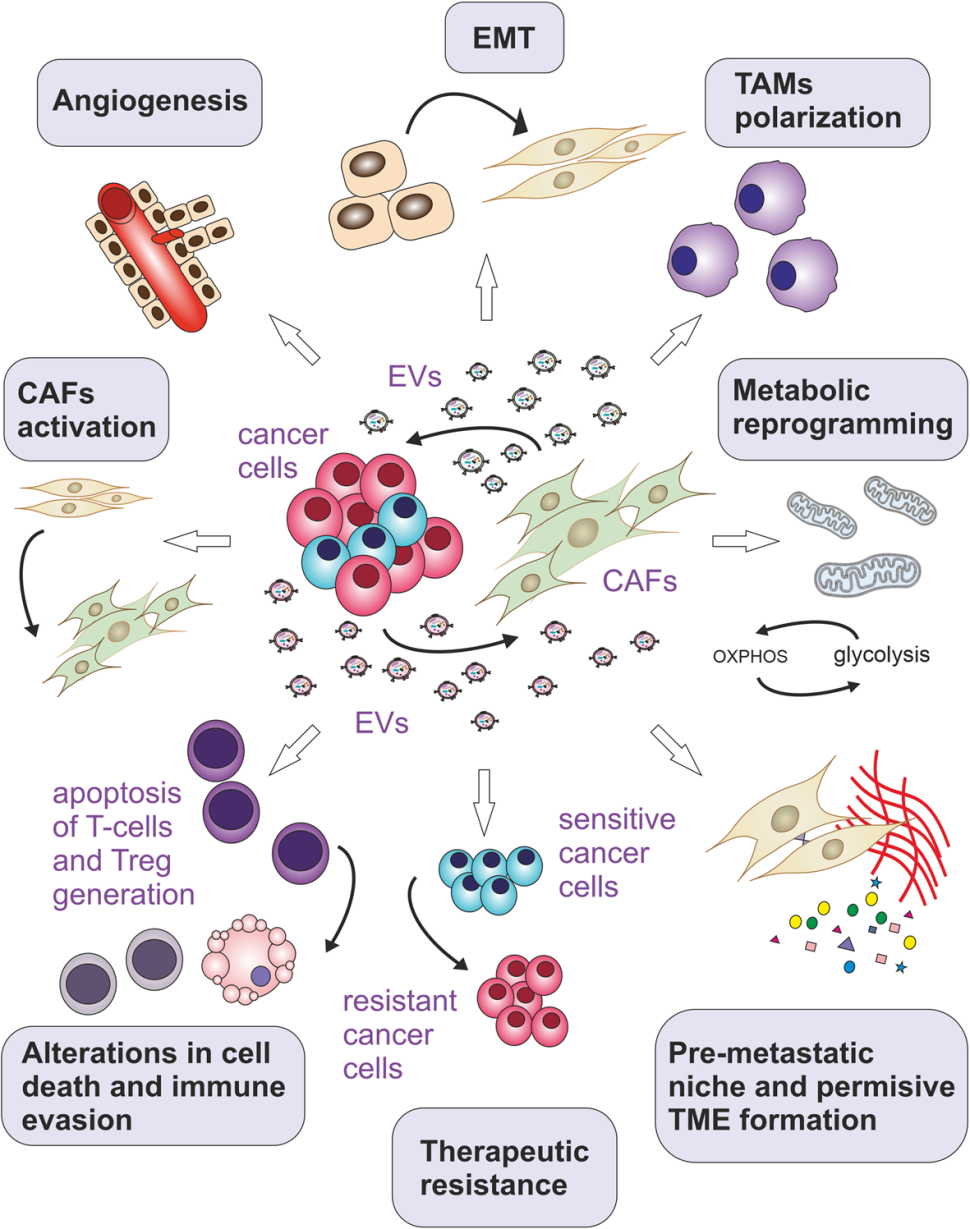
The role of exosomal proteins in specific hallmarks of cancer. Both stromal cell and cancer cell-derived exosomes are implicated in processes promoting cancer progression. These include epithelial-mesenchymal transition (EMT), angiogenesis, activation of cancer-associated fibroblasts (CAFs), immune evasion, polarisation of tumour-associated macrophages, insensitivity to cell death signals, pre-metastatic niche formation and metastasis, and even therapy resistance. Treg represents an immunosuppressive type of CD4 + T-cells. Treg can suppress anticancer immunity and cause immune evasion

Both tumour and stromal cell-derived exosomes are implicated in processes important for all stages of cancer progression, including tumour growth and cell proliferation, cell death avoidance, angiogenesis, immune evasion, invasion and metastasis, or even therapy resistance (see Fig. 2). Cancer cells secrete higher amounts of exosomes than normal cells, these exosomes are of altered composition. Several oncogenes and tumour suppressors have been found that are implicated in the regulation of exosomal biogenesis and production [99]. In addition, tumour cells can reprogram their metabolism in favour of glycolysis by enhancing the activity of glucose transporters. Increased glucose uptake leads to the elevation in lactate production through aerobic glycolysis and, thus, to intracellular accumulation of protons. This process is called the Warburg effect [100]. Accumulated protons are actively transported into the extracellular microenvironment via vacuolar ATPase (V-ATPase), Na^+^/H^+^ exchanger (NHE), monocarboxylate transporters (MCTs), and carbonic anhydrase (CAs) [101]. Elevated extracellular levels of acidic metabolites then lead to a lowering in extracellular pH. Low pH condition is considered one of the hallmarks of cancer, which can potentially influence exosome secretion and uptake [70, 102]. Moreover, exosomes released in acidic conditions (pH 6.0) were found to contain higher amounts of certain protein categories engaged in focal adhesion, actin cytoskeleton regulation, leukocyte migration through endothelia, or cell morphology modification. These molecules include H-Ras (Harvey rat sarcoma virus), N-Ras (Neuroblastoma RAS viral oncogene homolog), GANAB (glucosidase II alpha subunit), HSP90B1 (heat shock protein 90 beta family member 1), TIMP3 (tissue inhibitor of metalloproteinase-3) and other proteins, which are also linked to metastatic melanoma patient’s poor prognosis [70, 103].

As mediators of cell-to-cell communication, exosomes play a pivotal role in the tumour microenvironment (TME). Various bioactive molecules loaded to exosomes are necessary signals for reprogramming of TME in favour of cancerogenesis [104]. For example, delta-like 4 protein (DLL4) increases vessel branching and promotes cancer-associated modifications of the TME [105]. DLL4 was associated with tumour aggressiveness and an unfavourable clinical outcome in colorectal cancer patients [106]. Integrins are a family of proteins that direct exosomes to specific tissues, thus being partly responsible for premetastatic niche formation and metastatic tropism during breast cancer development [107]. Exosomal transforming growth factor-beta (TGFβ) triggers fibroblast and mesenchymal stem cell differentiation into myofibroblasts, promoting cancer proliferation and invasiveness, for example, in prostate cancer [108, 109]. Exosomal tetraspanin 8 (Tspan8) promotes angiogenesis in adenocarcinoma through increasing endothelial cell proliferation, migration and sprouting [110].

The TME is comprised of diverse cell types, such as endothelial cells, fibroblasts, and immune cells, with various functions, including impact on cancer development and progression [111]. Because of its heterogeneity and adaptability, the TME is partly responsible for therapy resistance [112]. The recruitment of immune cells into the TME is governed by dynamic signalling, part of which are exosomes [113]. Tumour-derived exosomes (TDEs) can directly influence the differentiation and activity of NK (natural killer) cells, macrophages, T-cells and B-cells [114]. Fibroblasts are an integral part of the TME. They play a critical role in maintaining homeostasis in connective tissues by producing extracellular matrix (ECM) components and various cytokines. Fibroblasts are usually in a quiescent state, as their levels of proliferation and metabolic activity are low [115]. In response to cancer cell presence, stromal fibroblasts can be activated into cancer-associated fibroblasts (CAFs) that can promote invasive growth and metastasis [116]. CAFs are characterised by morphological features, such as spindle shape and lack of expression of non-mesenchymal cell markers typical for epithelial, endothelial, immune, or neuronal cells [117, 118]. CAFs express specific protein markers, such as fibroblast activation protein α (FAP), α-smooth muscle actin (α-SMA), fibroblast-specific protein 1 (FSP1), podoplanin (PDPN), and platelet-derived growth factor receptor (PDGFR) [115, 119]. CAFs also produce a variety of proinflammatory and inflammation-activating factors, like nuclear factor kappa B (NF-κB), IL-6, FGF-2 (also known as bFGF), or TGFβ [120]. The mechanism of fibroblast activation is still not well understood. However, TDEs are believed to be important factors promoting CAF activation and proliferation, as they contain TGFβ and activate SMAD-dependent signalling [121, 122]. Following the interaction with TDEs, mesenchymal stem cells (MSCs) might also give rise to CAFs. Exosomes secreted from breast cancer cells (MCF-7, or MDA-MB-231 cells) induced the differentiation of adipose-derived mesenchymal stem cells into the myofibroblastic type of CAFs. This process is accompanied by the increased secretion of CAF-produced factors, such as vascular endothelial growth factor (VEGF), stromal cell-derived factor 1 (SDF-1), and TGFβ, which are engaged in tumour progression and metastasis regulation [123] as CAFs can contribute to the establishment of a pre-metastatic niche [124, 125].

Exosomal CD151 and Tspan8 are essential for cancer cells and CAFs communication with a contribution to ECM remodelling. TDEs derived from lung-tropic tumours express high levels of specific integrins, α6β1 or α6β4, which enable TDEs to bind to lung fibroblasts, leading to the formation of pre-metastatic niche in lung [107, 126]. Exosomes are key players not only in CAF activation but also in the crosstalk between CAFs and tumour cells because CAF-secreted exosomes can affect the tumour phenotype via their specific cargo [120]. These exosomes can fuel cancer cell metabolism by transporting various metabolites, like amino acids, lipids, Krebs cycle metabolites, or even mitochondria [127, 128].

### Angiogenesis

Angiogenesis is the process of forming new blood vessels, and it has a pivotal role in tumour progression to advanced stages of cancer (neovascularisation is necessary when the tumour volume exceeds 1 mm^3^) [129], including metastatic site formation. The formation of new vessels from pre-existing ones is induced by an imbalance between pro- and anti-angiogenic factors, VEGF overproduction, in particular. Cancer cells can obtain angiogenic phenotype in the process called angiogenic transition, which leads to uncontrolled production of proangiogenic factors and excessive angiogenesis. Manipulation of angiogenesis has become one of the approaches for cancer therapy, although its current efficacy is limited [130]. Specifically, bevacizumab is a widely used angiogenesis inhibitor for metastatic colorectal carcinoma therapy [131]. According to numerous studies, exosomes can accelerate angiogenesis via their cargo.

An important factor significantly involved in angiogenesis is TGFβ-I. The study on head and neck squamous cell carcinoma (HNSCC) cell lines reveals that TGFβ-enriched TDEs are major factors driving angiogenesis in the TME [132]. Surprisingly, TDEs promote TGFβ-signalling not only in endothelial cells but also in other cell types within the TME, such as macrophages. TDEs enriched in TGFβ promote the differentiation of non-activated macrophages into a tumour-associated macrophage-like (M2-like) phenotype, which is proangiogenic [133]. These TGFβ-enriched exosomes might be promising targets in anti-angiogenic therapy via blocking TGFβ interactions using mRER-mediated silencing. TGFβ signalling is blocked by neutralising extracellular TGFβ by mRER [134]. mRER is a newly developed TGFβ inhibitor that acts as a ligand trap for TGFβ and significantly inhibits angiogenesis [133]. Due to its high efficiency even in picomolar concentrations and small size, mRER enables better penetration of dense tissues, for example, the extracellular matrix [135].

The protein content of TDEs, including various pro-angiogenic factors, widely differs in distinct types of cancer. Specifically, glioblastoma-derived exosomes carry angiogenin, VEGF, TGFβ, IL-6, IL-8, and tissue inhibitor of metalloproteinase-1/2 (TIMP-1/2), which regulate MMP activity [136, 137]. In nasopharyngeal carcinoma, exosomes are highly enriched in intercellular adhesion molecule-1 (ICAM-1), CD44 variant isoform 5 (CD44v5) and matrix metalloproteinase 13 (MMP-13), in contrast, angio-suppressive protein TSP-1 is downregulated in these exosomes [138, 139]. Exosomes derived from multiple myeloma contain VEGF, bFGF, MMP-9, hepatocyte growth factor (HGF), and serpin E1 [140]. In lung adenocarcinoma, exosomes enriched in sortilin (also known as neurotensin receptor 3, NTSR3) might upregulate the level of expression of some pro-angiogenic proteins, namely endothelin-1, IL-8, thrombospondin-2 (TSP-2), uPA, and VEGF. Sortilin also promotes the release of TDEs themselves and may be useful as a diagnostic and prediction marker of cancer progression [141, 142]. Exosomal annexin II promotes angiogenesis in breast cancer by acting as a co-receptor for tissue plasminogen activator (tPA) [143]. In bladder cancer exosomes, EGF-like repeats and discoidin I-like domain-3 (EDIL-3), which is essential in angiogenesis and vascular development, is overexpressed [144].

As tumour grows, the demand for oxygen and nutrients increases. Existing blood vessels may not be sufficient to meet this demand and the formation of new blood vessels through neoangiogenesis may not always keep pace with the rapid growth of the tumour mass, leading to the persistence of hypoxic regions within the tumour. Hypoxia is one of the critical conditions responsible for the influence on exosome biogenesis, their release and content, hence on cancer progression. The adaptation of cells to reduced oxygen supplies is provided by hypoxia-inducible factors (HIFs) [145]. High expression of HIF-1α contributes to the heterogeneity of tumours and more aggressive phenotype [146]. The HIF-1 pathway acts as a key regulator of angiogenesis in both physiological (e.g., embryonic development, wound healing) and pathological (e.g., cancer, chronic inflammation) processes. HIF-1 works synergistically with other pro-angiogenic factors, namely VEGF, placental growth factor (PIGF), and angiopoietin 1/2 and upregulates their expression [147]. HIF-1 can be activated not only by hypoxia but also by genetic alterations in malignant cells that block HIF-1α ubiquitination, and therefore its proteasomal degradation [148]. Specifically, the deletion of tumour suppressor genes like *p53*, *p21*, *pRb*, or *PTEN* leads to HIF-dependent stimulation of VEGF and, thus, to angiogenesis stimulation [149]. Knowing that, HIF-1 seems to be a promising therapeutic target for many diseases linked to angiogenesis, including cancer [147]. Furthermore, HIF-1α induces exosome release via transactivating the small GTPase Rab22A in breast cancer [150]. Hypoxia-regulated exosome secretion in different tumours can be induced by an actin dynamics regulator RHO-associated protein kinase (ROCK), or calpain, which is responsible for membrane phospholipids asymmetry and membrane bending [151]. The hypoxic state causes a higher secretion of TDEs of altered content [152]. Alterations in exosome cargo under hypoxic conditions mediate tumour microenvironment remodelling and promote tumour progression, including immune evasion, angiogenesis, metastasis, and therapy resistance. The key proteins loaded to hypoxic exosomes include, for example, HIF-1α (nasopharyngeal carcinoma) [153], lysyl oxidases, PDGFs, thrombospondin-1 (TSP-1), plasminogen activator inhibitor 1 (PAI1), caveolin-1 (all in glioblastoma) [154–156], annexin II (prostate cancer) [157], or signal transducer and activator of transcription 3 (STAT3) (ovarian cancer) [152].

### Cell death and proliferation

The replicative immortality of cancer cells is often accompanied by an altered sensitivity to regulated cell death (RCD), which includes apoptosis, necroptosis, ferroptosis, and pyroptosis [158]. RCD encompasses the organised demise of cells under the control of specific genes and molecular pathways, aiming to uphold homeostasis. Cancer is, among others, characterised by dysregulated cell death and increased proliferation of cancer cells. Typically, cancer cells sustain proliferative activity through the activation of the PI3K/AKT (phosphatidylinositol 3-kinase/protein kinase B, also PKB) or MAPK/ERK (mitogen-activated protein kinase/extracellular signal-regulated kinase) pathways. These pathways can be directly activated by TDEs. For example, in NSCLC, bladder and prostate cancer, PI3K/AKT is activated, while in gastric cancer, both the PI3K/AKT and the MAPK/ERK signalling pathways are activated by TDEs [159]. TDEs are also able to support evasion from growth suppression. However, the role of TDEs in this process is less defined. For example, TDEs could downregulate growth suppression via delivering exosomal oncogenic H-Ras and N-Ras transcripts or Rab proteins [160]. The replicative immortality can be obtained by the upregulation of telomerase, which is stimulated by various transcription factors and coregulators. Factors that significantly modulate telomerase activity, such as c-Myc, p53 and β-catenin, are known to be TDE cargo, but a direct role of exosomes in telomerase activation is not known [159].

With ongoing research, it is becoming increasingly apparent that EVs possess the ability to modulate cell death responses in recipient cells. These EVs can be either derived from living cells or from apoptotic cells themself. For example, exosomes with membrane-bound TNF-α produced by fibroblasts can inhibit activation-induced cell death (AICD) in CD4^+^ T-cells [161]. Similarly, AICD of T-cells can be triggered by Fas ligand-bearing exosomes [162]. Exosomes derived from N-myc amplified neuroblastoma cells enhance the survival of non-N-myc amplified cells by inducing chemoresistance to doxorubicin [163]. On the other hand, colorectal cancer cells can induce apoptosis of T-cells through the release of proapoptotic microvesicles [164]. Additionally, TDEs can contain inhibitors of apoptosis (IAP) such as survivin, XIAP, cIAP1 or cIAP2 [165], which can inhibit apoptosis in cancer cells. Bladder cancer TDEs were shown to inhibit apoptosis through the upregulation of Bcl-2 and cyclin D1, and Bax and caspase-3 downregulation in target cells [166].

A newly discovered group of EVs released during apoptosis, called apoptotic exosomes (ApoExos), may represent a significant player in communication and signalling in the TME. ApoExos are implicated in diverse pathophysiological processes, including vascular homeostasis, sterile inflammation, as well as proliferation and survival of cancer cells [167], and are released as a consequence of pre-apoptotic stress or post-apoptotic necrosis [168]. Autolysosomes were also identified as a site of ApoExos biogenesis, and caspase-3 as a key regulator of the secretion of various types of EVs, including ApoExos [169]. In glioblastoma, a highly aggressive brain cancer, it has been discovered that specific components of the spliceosomes present in ApoExos facilitate tumour cell proliferation and confer resistance to therapy [170]. ApoExos widely express exosomal marker CD63, lysosomal marker LAMP1 (lysosomal associated membrane protein 1), and HSP70, which is commonly expressed under apoptotic conditions [167]. A crucial role in regulated cell death, apoptosis and pyroptosis in particular, is played by a group of cysteine aspartic proteases known as caspases (casp), classified as apoptotic (casp-3/6/7/8/9) and inflammatory (casp-1/4/5/12) [171]. Caspases play a part in EV biogenesis, cargo loading and processing and can also be loaded into exosomes. For example, casp-3 dependent intra-vesicular cleavage of Bcl-xL (B-cell lymphoma-extra-large) is required for the uptake of EVs by malignant blood cells. Casp-3 inhibition then results in reduced cell proliferation of recipient tumour cells [172]. Targeting caspases as novel anticancer therapy is being currently developed with a focus on small drugs or gene editing [173].

Necroptosis is defined as a regulated form of cell death accompanied by inflammation. The activation of necroptosis could also mediate the immune escape of cancer cells and the rise of metastasis through the attraction of tumour-associated macrophages, for instance, in pancreatic cancer cells by releasing CXCL5 (C-X-C motif chemokine 5) [174, 175]. Surprisingly, necroptotic cells are also able to release EVs loaded with various cargo, including proteome unique for necroptotic EVs. Specifically, casp-8, mixed lineage kinase domain-like kinase (MLKL), charged multivesicular body protein 4B (CHMP4B), ESCRT-III components, several Rab proteins (Rab5A/B/C), SNAREs, flotillin-1/2, and lipid-raft-associated proteins are detected in higher levels in necroptotic EVs. In addition, necroptotic EVs induce the secretion of pro-inflammatory molecules, such as IL-6, TNF-α, and CCL2 (C–C motif chemokine ligand 2) [176, 177]. Since many cancers are associated with a decrease or absence of necroptotic factors, which leads to necroptosis resistance, necroptosis appears to be a promising target for cancer therapy [177]. TDEs preferentially target malignant cells, as will be discussed later, and can also be engineered to start the necroptosis pathway. A novel therapeutic strategy proposed a method for CRISPR/Cas9 delivery via exosomes. In principle, two CRISPR/Cas9 vectors might target and inactivate IAP1/2 (inhibitor of apoptosis protein 1/2) and casp-8, resulting in necroptosis activation [178].

Another form of RCD, ferroptosis, is characterised by an iron-dependent accumulation of lipid hydroxyperoxides and is regulated by glutathione peroxidase 4 (GPX-4). The intracellular iron homeostasis contributes to ferroptosis sensitivity in diverse cells. Since free iron levels vary between various stages of cancer (metastatic vs. non-metastatic cells), differences in ferroptosis sensitivity are expected. To set an example, breast cancer cells can resist ferroptotic cell death by upregulating iron export out of the cell, for instance, via exosomes. This resistance can be suppressed by a blockage of prominin 2, a pentaspanin protein that promotes MVBs formation, and thus iron secretion via exosomes [179]. In the TME, ferroptosis drives macrophage polarisation and thus promotes tumour growth. Specifically, extracellular protein K-ras was found to be packed into TDEs that are uptaken into macrophages. K-ras uptake leads to a switch from M1 to M2 macrophage phenotype and cancer progression. Similarly to necroptosis, ferroptosis-inducing components, such as erastin, can be loaded into exosomes to target cancer cells [180]. For instance, imidazole ketone erastin (IKE) reduced tumour growth by inducing ferroptosis in a diffuse large B cell lymphoma (DLBCL) mouse xenograft model [181].

### Immune system, inflammation, and immune evasion

Immune evasion in cancer refers to the ability of cancer cells to evade or escape recognition and attack by the immune system. Inflammation and immune evasion are interconnected processes that play crucial roles in cancer development and progression. For example, chronic inflammation can suppress cytotoxic T-cell activity and was associated with tumour progression [182, 183]. TDEs are known to be strongly involved in these immunomodulatory processes. Cytokines, small proteins, which are key mediators of immunity and inflammation, are extensively associated with exosomes. These cytokines include IL-1α, IL-1β, IL-6, IL-8, IL-18, TNF-α, COX-2 (cyclooxygenase 2), VEGF, CCL2, CCL3, CCL4, CCL5, CCL20 [184, 185]. The proinflammatory response may also be stimulated by HSP70, which is elevated in cancer exosomes. HSP70 enriched exosomes can trigger NF-κB activation and TNF-α release or increased IFN-γ and IL-13 production [185, 186]. The group of aminoacyl-tRNA synthetases (ARSs) is also involved in immune and inflammatory modulation, angiogenesis, or apoptosis if it occurs extracellularly. Specifically, lysyl-tRNA synthetase (KRS) is secreted via colorectal cancer cell-derived exosomes, which induces proinflammatory cytokines production [187].

Tumour cells are capable of escaping the immune system reaction with the help of tumour-infiltrating regulatory T-cells (Tregs) by releasing immunosuppressive cytokines, namely IL-10 and TGFβ1 [188]. TDEs enable Treg generation and expansion, thus promoting cancer progression. Moreover, Th17-cells (CD4^+^ T-lymphocytes secreting essential amounts of IL-17A [189]) might also induce immunosuppression and angiogenesis to facilitate tumour progression, or they can recruit immune cells to promote antitumour immune response [190]. The role of Th17 in the context of tumour growth depends on the ratio of Treg/Th17 [191]. TDEs also highly express tumour antigens on their surface. This characteristic has led to the suggestion that they could serve as tumour vaccines. However, they can also suppress T-cell signalling molecules and induce apoptosis in T-lymphocytes. For instance, ovarian cancer cell-derived exosomes exhibit FasL to suppress the immune response by inhibiting T-cells and inducing their apoptosis [192].

TNF-α is a pro-inflammatory cytokine mainly produced by macrophages, NK cells, and T-cells, but also by non-immune cells like fibroblasts, endothelial cells, and neurons. TNF-α exhibits a dual role in relation to cancer. On the one hand, the anticancer property of TNF-α is to induce cancer cell death, but on the other hand, TNF-α also stimulates cell proliferation, migration and angiogenesis and is highly overexpressed in many cancers [193, 194]. Since TNF-α is often present in TDEs, for example, in colorectal carcinoma, there may be a beneficial effect of decreasing TNF-α in anti-cancer therapy [195]. Natural killers (NK) are a group of cells involved in antitumour immune response via the natural killer group 2 member D (NKG2D) activating receptor [196]. Loss of the NKG2D receptor or its function leads to immune evasion. TDEs can contribute to NK-cells activity suppression by the expression of NKG2D ligands, which depress NKG2D receptors on NK-cells and inhibit their cytotoxicity [197].

In addition, tumour-derived exosomes are known to inhibit the maturation and differentiation of monocytes (monocytes give rise to macrophages and dendritic cells), and consequently induce immunosuppression. The mechanism of immunosuppression might be dependent on the protein composition of exosomes, namely TGFβ, IL-6, or prostaglandin E2 (PGE2) [198]. To set an example, the secretion of IL-6, which functions in the PI3K/AKT/mTOR pathway [199], by TDEs inhibits the differentiation of bone marrow myeloid precursors into DCs. DC maturation can also be affected by the intake of exosomal TGF-β1 by immature DCs [200]. Furthermore, TDEs can influence macrophages to switch into polarised M2 phenotype. For example, TDEs derived from triple-negative breast cancer (TNBC) play role in M2 macrophages polarisation, which benefits tumour growth and lymph-node metastasis formation [201]. M2 macrophages then secrete high amounts of cytokines, growth factors and enzymes, including already mentioned VEGF, PDGF, TGFβ, and some MMPs, that facilitate immunosuppression, angiogenesis, metastasis, or treatment resistance [202, 203]. Monocytes that fused with TDEs possess an immunosuppressive effect, which leads to a high CD14 expression. CD14^+^ monocytes (but without HLA-DA expression) were proven to be increased in the serum of many cancer patients as tumour-induced immunosuppressors [200]. To promote their growth and proliferation, tumours also respond to endoplasmic reticulum stress (ER stress), which helps them evade the immune system recognition and response. ER stress also increases the production of pro-inflammatory factors in macrophages and modifies immune cell function [204]. A study on oral squamous cell carcinoma (OSCC) revealed that macrophage polarisation toward the M2 subtype is promoted by programmed death ligand 1 (PD-L1) enriched exosomes derived from ER-stressed cancer cells. PD-L1 overexpression was linked to the poor overall survival of OSCC patients [205, 206]. PD-L1 expression can also be enhanced in acidic TME [207]. Macrophages can also be switched to the M2 phenotype by high lactic acid and the hypoxic environment through the expression of arginase 1 (ARG1) [208].

Furthermore, myeloid-derived suppressor cell (MDSC) accumulation negatively affects antigen processing and presentation and produces immunosuppressive factors. This function of MDSCs is potentiated by TDEs [200]. In renal cancer, exosomal HSP70 enhances MDSC activation via activating TLR2 signalling [209]. Next, TGFβ and PGE2 in exosomes isolated from breast cancer help to accumulate MDSCs, thus enhancing tumour growth [210]. Exosomes derived from OSCC cells under hypoxic conditions enhanced the immunosuppressive function of MDSCs to interfere with the group of *γδ* T-cells via miR-21/PTEN/PD-L1 signalling [211].

### Pre-metastatic niche formation, invasion, and metastasis

The formation of pre-metastatic niches, invasion, and metastasis are critical steps of cancer progression and are responsible for the widespread dissemination of cancer cells throughout the body. Exosomal proteins participate in various pro-metastatic mechanisms, including invasive behaviour promotion, induction of tumour neovascularisation, disrupting vascular barrier, mediating specific organ colonisation, and setting pre-metastatic niches. Tumour-derived exosomes participate in permissive niche formation, supporting the “seed and soil” hypothesis. This concept was introduced by Stephen Paget [212], who proposed that tumour cell (seed) growth requires the appropriate local microenvironment (soil). Although circulating tumour cells (CTCs) can be found in the vasculature of multiple organs, they do not necessarily give rise to metastasis. However, in advance of tumour cell dissemination, primary tumours can appropriate secondary sites, creating a pre-metastatic niche, which facilitates subsequent colonisation of this location by tumour cells [213, 214]. This is provided by the systemic signalling of tumour cells, including exosome secretion [215].

The essential TDEs-carried molecules for ECM remodelling and epithelial-mesenchymal transition (EMT) are TGF-β, HIF1α, β-catenin, IL-6, caveolin-1, or vimentin [216]. Tumour-derived EGFR (epidermal growth factor receptor)-containing exosomes are capable of remodelling the liver microenvironment presenting a novel mechanism concerning liver-tropism of gastric cancer metastasis [79]. Hepatocellular carcinoma (HCC) and breast cancer are known to metastasise in bone, which causes fractures due to osteolytic bone destruction. To survive, metastasised cells interfere with normal bone remodelling through the suppression of bone formation and activation of bone resorption. This is enabled by osteoclast differentiation, which leads to the release of bone-derived factors that support tumour growth [217]. HCC-derived exosomes were highly enriched in Tumour necrosis factor-α (TNF-α), which promotes osteoclast differentiation. TNF-α also regulates hepatocyte proliferation in liver cancer under uncontrolled inflammation [218]. To conclude, primary TDEs contribute to tumour metastasis by educating the primary and distant soil [219]. Moreover, exosomes also play a crucial role in the metastatic process by recruiting mesenchymal stem cells (MSCs) or regulating nutrient availability in the TME. MSCs associated with the tumour-like phenotype undergo morphological and structural changes due to TDE induction. The tumour-like phenotype includes atypical microvilli, pseudopods, higher vesicle secretion, and other changes, such as higher proliferation, invasive potential, and migration. Furthermore, proteins loaded in exosomes directly identify organs that are suitable for metastatic site formation [124]. Exosomal proteins not only promote tumour growth and metastasis but also serve as early markers of disease, as they are easily accessible for clinical detection and highly secreted in cancer patients [37]. HSP70 was found to be expressed in the membranes of TDEs, in contrast to normal cells. Levels of HSP70-enriched exosomes are also increased in metastatic patients compared to non-metastatic patients or healthy individuals, thus, HSP70 may be used as a biomarker of cancer progression [220].

In breast cancer exosomes, fibronectin is involved in promoting metastasis via EMT and production of pro-inflammatory cytokines and MMP-9 [221], metastasis-associated protein 1 (MTA1) is linked to enhanced metastatic potential and unfavourable prognosis in breast cancer patients [222]. Exosomal TSP1 mediates the disruption of endothelial cell integrity and the reduction of junction proteins VE-cadherin and ZO-1 expression, thus, facilitating trans-endothelial migration of breast cancer cells [221, 223]. A study on mice with breast cancer revealed that exosomal nephronectin (NPNT) regulates the ability of breast cancer cells to colonise lung [224]. Cell migration-inducing and hyaluronan-binding protein (CEMIP) from brain metastatic cell-derived exosomes contributes to brain tumour invasion and association with brain vasculature, leading to enhanced tumour growth. In addition, CEMIP induces pro-inflammatory cytokines secretion, such as chemokines coded by *CCL/CXCL* genes or the *TNF* superfamily, thus promoting metastasis [225]. S100 calcium-binding protein A4 (S100A4) plays a pivotal role in tumour metastasis by regulating ECM remodelling, cellular adhesion, and motility. S100A4 identified in highly metastatic HCC exosomes promotes metastasis via the phosphorylation of STAT3 and upregulation of osteopontin, a typical HCC promoter [226]. In pancreatic cancer cell-derived exosomes, proteins CXCR4 (C-X-C motif chemokine receptor 4) and MMP-9 were found to enhance the metastatic capabilities of pancreatic cancer cells [227]. Similarly, enhanced secretion of exosomal MMP-1 promotes tumour cell invasion in gastrointestinal stromal tumours (GIST). The oncogenic protein tyrosine kinase (KIT)-containing exosomes trigger the conversion of progenitor smooth muscle cells to tumour-like phenotype and mediate the release of MMP-1 [228].

We have mentioned so far that cancer cells are able to take advantage of exosomal protein cargo by its uptake, however, they can also decrease intracellular levels of unwanted proteins or tumour-suppressors via exosome secretion. For example, metastatic duodenal carcinoma cells (AZ-P7a) do not tolerate intracellular accumulation of polyadenylate-binding protein 1 (PABP1). Consequently, PABP1 was found to be highly enriched in metastatic duodenal carcinoma (AZ-P7a) derived exosomes compared to normal cells (AZ-521) [229]. Furthermore, ST6Gal 1(beta-galactoside alpha-2,6-sialyltransferase)-depleted colorectal cancer cells remove tumour-metastasis suppressor kangai 1 (KAI1, also known as CD82) via exosomes as a mechanism to enhance metastatic formation [124, 230]. Stimulator of interferon genes (STING), which serves as an adaptor protein in the innate immune response to DNA damage or virus infection, can also be translocated into EVs through interaction with signal transducing adapter molecule (STAM). The translocation of STING into EVs served for STING degradation. EV-secreted STING downregulated the innate immune response [231].

### Cancer treatment and therapy resistance

The effectiveness of cancer screening, as well as successful early diagnosis and accurate risk assessment for cancer, are highly dependent on the specificity and quality of the biomarkers used. Many studies suggest that TDEs may be very promising cancer biomarkers. In recent years, exosomes were widely investigated in clinical trials (listed on ClinicalTrials.gov (https://clinicaltrials.gov/) with applications as biomarkers, drug-delivery systems, cancer vaccines, or exosome-based therapies. ClinicalTrials.gov includes 116 studies, of which 58 (50%) have been involved in studies of exosome biomarkers and 74.13% of those 58 trials were in relation to cancer. Another 33 studies (28.44%) have been registered for exosome-based therapy, most of them were focused on exosomes derived from MSCs. Overall, 6 studies (5.17%) have been registered for drug-delivery systems, and 2 clinical trials (1.72%) for exosome-based vaccines. The remaining 17 trials (14.66%) have been focused on basic analysis [232]. Here we present clinical trials from ClinicalTrials.gov focussed on exosomal protein content as a potential biomarker in relation to cancer (Table 1).

**Table 1:** Clinical trials focused on exosomal proteins as cancer biomarkers

Number	Status	Cancer type	Exosomal content
NCT01840306	Completed	HER2 + Breast Cancer	Not specified
NCT05463107	Not yet recruiting	Follicular Thyroid Cancer	thyroglobulin, Gal-3, calprotectin A8/A9, keratin 8/19, afamin, angiopoietin-1
NCT02862470	Completed	Anaplastic Thyroid Cancer	thyroglobulin, Gal-3 [233]
NCT04529915	Active	NSCLC	Not specified
NCT05735704	Recruiting	Haematological malignancies	Not specified
NCT03581435	Unknown	Gallbladder Carcinoma	Protein profile
NCT03985696	Recruiting	Non-Hodgkin B-cell Lymphomas	CD-20, PD-L1

*Gal-3* galectin-3, *NCT* National Clinical Trials, *PD-L1* Programmed death-ligand 1, *CD* cluster of differentiation

Alterations in protein or nucleic acid content of exosomes in plasma strongly correlate with pathological states of many diseases, including cancer even in early stages. Each millilitre of human blood contains over 10^9^ exosomes; thus in vivo detection of exosomes is highly sensitive [37, 234, 235]. The summary of exosomal proteins with potential for clinical diagnostic applications of various types of cancer is listed in Table 2.

**Table 2:** Exosomal proteins with potential for cancer diagnostics

Protein marker	Cancer type	Fluid	Diagnostic efficiency	Reference
Panel of 17 exosomal proteins^a^, TMEM256	Prostate cancer	Urine	Sensitivity 100%, specificity 60–100% (highest TMEM256)	[236–238]
Del-1	Breast cancer	Plasma	Sensitivity 94.70%, specificity 86.36%	[239, 240]
CA125	Ovarian cancer	Plasma	Sensitivity 71%, specificity 98%	[241–244]
GPC-1	Pancreatic cancer	Peripheral blood, Serum	Sensitivity 100%, specificity 100%	[244–247]
c-Met	Pancreatic cancer	Serum	Sensitivity 70%, specificity 85%	[238, 248]
GKN-1	Gastric cancer	Serum	Sensitivity 91.2%, specificity 96.0%	[249, 250]
L1CAM	Gastric cancer	Serum	Sensitivity 83.1%, specificity 62.2%	[251]
Panel of 6 exosomal proteins^b^	Colorectal cancer	Tissue	Sensitivity 70–100%, specificity 70–100%	[252]
CAE	Colorectal cancer	Serum	Sensitivity 89.47%, specificity 95.88%	[253, 254]
LG3BP	Hepatocellular carcinoma	Serum	Sensitivity 96.6%, specificity 71.8%	[238, 255, 256]
PIGR	Hepatocellular carcinoma	Serum	Sensitivity 82.8%, specificity 71.8%	[238, 255, 256]
TACSTD2	Bladder cancer	Urine	Sensitivity 73.6%, specificity 76.5%	[238, 257]
CD151	Lung cancer	Plasma	Sensitivity 60%, specificity 75%	[37, 258]
CD91	Lung cancer	Serum	Sensitivity 60%, specificity 89%	[238, 259]
AHSG	NSCLC	Serum	Sensitivity 54.9%, specificity 84.8%	[89]
CXCL7	OSCC	Serum	Sensitivity 60%, specificity 80%	[260]
BATF2	Nasopharyngeal carcinoma	Serum	Sensitivity 81%, specificity 82%	[261, 262]
PD-L1	Melanoma	Plasma	Sensitivity 80%, specificity 89.47%	[84]
Caveolin-1	Melanoma	Plasma	Sensitivity 69.0%, specificity 96.3%	[263]
EGFR VIII	Glioblastoma	Plasma	Sensitivity 68%, specificity 100%	[137, 238, 264]

*Del-1* developmental endothelial locus-1, *GPC-1* glypican-1, *c-Met* proto-oncogene mesenchymal-epithelial transition factor, *GKN-1* gastrokine 1, *L1CAM* L1 cell adhesion molecule, also CD171, *CAE* carcinoembryonic antigen, *LG3BP* galectin-3-binding protein, *PIGR* polymeric immunoglobulin receptor, *TACSTD2* tumor-associated calcium signal transducer 2, *AHSG* alpha-2-HS-glycoprotein, *CXCL7* C-X-C motif ligand 7, *BATF2* Basic Leucine Zipper ATF-Like Transcription Factor 2

^a^Including: TMEM256 (transmembrane protein 256), ADIRF (adipogenesis regulatory factor), LAMTOR1 (late endosomal/lysosomal adaptor and mitogen-activated protein kinase 1), plastin-2, several Rab-class members (e.g. Rab-2A, Rab-3B, Rab-3D, Rab-7A, Rab-6A), VALT (V-type proton ATPase 16 kDa proteolipid subunit), STEAP4 (Six-transmembrane epithelial antigen of prostate 4), DJ-1 (protein deglycase/ Parkinson disease protein 7), S100-P, synaptotagmin-like protein 4, ADP-ribosylation factor-like protein 8B, proton myo-inositol cotransporter, tetraspanin-6

^b^Including: NHP2 (H/ACA ribonucleoprotein complex subunit 2), OLFM4 (olfactomedin-4), TOP1 (DNA topoisomerase 1), SAMP (serum amyloid P-component), TAGL (transgelin), TRIM28 (tripartite motif-containing protein 28)

Another great potential of exosomes lies in their use as drug carriers. As mentioned before, exosomes protect their content with the lipid bilayer, and they can easily enter recipient cells. In addition, as exosomes are native to the organism, they do not cause any major side effects [265]. The ability of exosomes to serve as drug carriers was proven in many studies, for example, cisplatin-loaded exosomes extended the survival time of mice with ovarian cancer [266], macrophage-derived exosomes with paclitaxel inhibited Lewis lung cancer cells proliferation, and even possess better stability of loaded paclitaxel than other loading approaches [267]. Adding more, since tumour cells frequently communicate via exosomes, TDEs may also deliver therapeutic drugs to other tumour cells. For instance, prostate cancer-derived exosomes loaded with paclitaxel can be uptaken by prostate cancer cells [268], similarly, exosomes from pancreatic cancer cells can deliver curcumin and induce cell apoptosis in pancreatic cancer cells [269]. Many preclinical studies on the role of exosomes as therapeutic drug carriers for cancer therapy have already been assessed, but more must be investigated [90]. Another promising therapeutic approach might be targeting cancer exosome release itself. Exosome secretion is mediated by an intracellular increase of calcium (Ca^2+^), which is regulated by the H^+^/Na^+^ and Na^+^/Ca^2+^ channels [270]. Blocking these channels with dimethyl amiloride (DMA), for example, reduced the amount of secreted exosomes in mice with colon carcinoma (CT26) [271].

Monoclonal antibodies (mAbs) are used in cancer immunotherapy to stimulate the function of the immune system and enhance the targeting of conventional anticancer drugs. Upon binding to tumour-associated antigens (TAAs), mAbs can disrupt crucial pathways that play a significant role in cancer cell activity. Nevertheless, TDEs carry several TAAs, therefore, they can decrease the efficacy of mAbs [272] as TAAs can bind antibodies used against cancer cells, which results in insufficient amounts of antibodies that can reach cancer tissue. For example, exosomes secreted from cancer cells reduce the therapeutic activity of trastuzumab (HER2 blocker, normally activates Ab-dependent cell-mediated cytotoxicity) in breast cancer therapy [273]. On the other hand, TDEs can represent an attractive alternative source of TAAs for cell-free cancer vaccines for personalised tumour immunotherapy [274]. TDEs can transfer TAAs to antigen-presenting dendritic cells (DCs). Some studies [275] showed that TDEs promote DC maturation and enhance antigen cross-presentation more potently than tumour cell lysates, which directly contributed to a more robust tumour-specific response of cytotoxic T-lymphocytes. Consequently, DCs treated by TDEs have the potential to effectively reverse immunosuppression in the TME. There have also been developed cell-free tumour vaccines containing α-fetoprotein-enriched DC-derived exosomes, which stimulate immune cells to produce IFN-γ and IL-2 and reduce the expression of TGFβ and IL-10 at the site of the tumour, thus, inducing antigen-specific response to cancer cells. This led to tumour growth inhibition and metastatic ability limitation [276].

Moreover, exosomal PD-L1 might represent a promising therapeutic target. It has been shown that metastatic cancer cells produce a high level of exosomes, that carry PD-L1 on their surface. PD-L1 then binds the PD-1 receptor on T-cells leading to the suppression of T-cell activity [84]. Blockade of PD-L1 can possibly bypass the current resistance to antibody therapies [277]. PD-L1 and CTL-associated antigen 4 (CTLA-4) serve as checkpoint receptors that are targeted for relieving exhaustion of CD8 T-cells caused by immunosuppression within the TME [278]. In addition, more possible targets may be relevant in this treatment strategy, namely T-cell immunoglobulin- and mucin-domain-containing molecule 3 (Tim-3), and its ligand galectin-9 [279]. Additionally, immune cell-derived exosomes can also participate in adoptive cell therapy (ACT), immunotherapy based on redelivering tumour-infiltrating lymphocytes (TILs) [280].

## Concluding remarks

As we discussed, exosomes are involved in many critical steps of cancer progression, including ECM remodelling, angiogenesis, immune regulation, invasion, metastasis, and therapy resistance, and their content plays a pivotal role as a signalling hub in the tumour microenvironment. Exosomal cargo is protected from enzymatic degradation because it is encapsulated within the lipid bilayer of exosomes, allowing exosomal proteins to retain their native conformation and functionality. Specific proteins loaded to exosomes not only reflect the proteome of the cell of origin but also serve as markers of the pathological state of the cell. As the exosomal content varies depending on the cell of origin, exosomes may be used as specific biomarkers that can provide information about the genetic and molecular heterogeneity of tumours. Exosomes play a significant role in intercellular communication, and their content can provide valuable information for cancer diagnosis, prognosis, and treatment (clinical studies dealing with exosomal proteins are listed in Table 1). Moreover, exosomal uptake by certain cells in the TME can be applied to cancer therapy, as exosomes can be loaded with various treatment drugs. The quantity of exosomes in the bloodstream or other body fluids may also indicate the stage and aggressiveness of cancer, as higher levels of exosomes may be associated with advanced disease. Alterations in tumour cell metabolism and decreased pH conditions within the TME promote TDE secretion.

Exosomes can serve as a source of “liquid biopsy” material, which can replace or complement traditional tissue biopsies. This non-invasive approach is suitable for regular testing and monitoring and is particularly useful for patients for whom invasive procedures are not possible. Exosomes find primary clinical utility as biomarkers, cell-free therapeutic agents, vehicles for drug delivery, and as a component in cancer vaccines. Ongoing research continues to uncover their specific roles and applications in different cancer types, bringing us closer to more effective and personalized approaches to managing cancer. While exosomes hold great promise, challenges include standardizing isolation and analysis techniques, as well as distinguishing between exosomes from cancer cells and those from non-malignant cells.

## Data Availability

Not applicable.
